# Turbulence in a matter-wave supersolid

**DOI:** 10.1038/s41598-018-30852-5

**Published:** 2018-08-22

**Authors:** C.-H. Hsueh, Y.-C. Tsai, T.-L. Horng, M. Tsubota, W. C. Wu

**Affiliations:** 1 Department of Physics, National Taiwan Normal University, Taipei, 11677 Taiwan; 2 Department of Applied Mathematics, Feng Chia University, Taichung, 40724 Taiwan; 3 Department of Physics, Osaka City University, Sugimoto 3-3-138, Sumiyoshi-ku, Osaka, 558-8585 Japan

## Abstract

Quantum turbulence associated with wave and vortex dynamics is numerically investigated for a two-dimensional trapped atomic Rydberg-dressed Bose-Einstein condensate (BEC). When the coupling constant of the soft-core interaction is over a critical value, the superfluid (SF) system can transition into a hexagonal supersolid (SS) state. Based on the Gross-Pitaevskii equation approach, we have discovered a new characteristic *k*^−13/3^ scaling law for wave turbulence in the SS state, that coexists with the waveaction *k*^−1/3^ and energy *k*^−1^ cascades commonly existing in a SF BEC. The new *k*^−13/3^ scaling law implies that the SS system exhibits a negative, minus-one power energy dispersion (*E* ~ *k*^−1^) at the wavevector consistent with the radius of the SS droplet. For vortex turbulence, in addition to the presence of the Kolmogorov energy *k*^−5/3^ and Saffman enstrophy *k*^−4^ cascades, it is found that large amount of independent vortices and antivortices pinned to the interior of the oscillating SS results in a strong *k*^−1^ scaling at the wavevector consistent with the SS lattice constant.

## Introduction

Turbulence in a superfluid (SF), named quantum turbulence (QT), has recently attracted considerable interest in both liquid helium^1–5^ and atomic Bose-Einstein condensates (BEC)^6–16^. Atomic BEC is a clean system with the advantage of easy manipulation, and thus is a good candidate for studying the QT. In a typical SF atomic BEC, turbulence mostly associated with *vortices* can be characterized by an *incompressible* energy spectrum following the Kolmogorov *k*^−5/3^ power law^17^, which describes the process wherein, if far away from forcing and sink, energy is transported conservatively to smaller scales^18^. In addition to energy, there could exist another conserved quantity in two dimensions (2D), named enstrophy, in association with the vorticity. Consequently in a 2D atomic SF BEC, in addition to the Kolmogorov *k*^−5/3^ energy-cascade spectrum, the vortex turbulence (VT) also consists of the Kraichnan *k*^−3^ (in case of strong anisotropy) or Saffman *k*^−4^ (in case of weak anisotropy) enstrophy-cascade spectrum^19–27^.

In addition to VT, turbulence consisting of *waves*, named wave turbulence (WT), also plays a central role in a system of interactions. As a general phenomenon, WT is observed in a vast range of nonlinear systems, on quantum to astrophysical scales^28^. In a 2D atomic SF BEC^29^, WT scaling laws typically involve the *k*^−1/3^ waveaction-cascade and the *k*^−1^ *compressible* energy-cascade spectra^29^. The former corresponds to the strongly nonequilibrated process of evaporative cooling and the latter corresponds to the condensation process, respectively.

**Figure 1:**
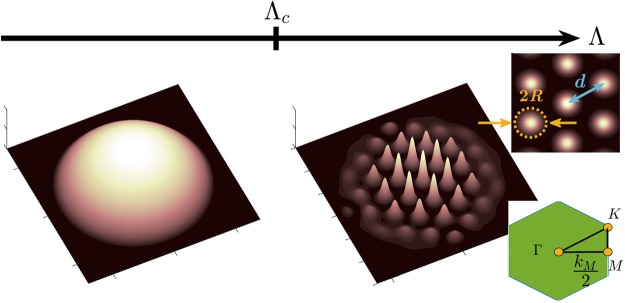
Schematic plot of a SF state transition into a hexagonal SS state in a quasi-2D trapped Rydberg-dressed BEC when the coupling constant Λ is increased from below to above a critical value Λ_*c*_. Right upper inset shows in the SS state that two new length scales, the lattice constant *d* and the radius of the SS droplets *R*, emerge. Right lower inset shows the first Brillouin zone of a 2D hexagonal lattice with the wavevector *k*_*M*_ defined.

Supersolid (SS) is a state of matter that simultaneously possesses superfluidity and solidity and in which both gauge and continuous translational symmetries are broken^30–34^. Recently SS states have been realized in a BEC coupled to the modes of two optical cavities^35^ and in a BEC with spin-orbit coupling^36^. Another promising candidate for the SS state is the Rydberg-dressed atomic BEC that exhibits a defocusing soft-core interaction^37–41^. In such a system, the interaction between the Rydberg-dressed ground-state atoms behaves as: $U(\bar{{\bf{r}}})={\mathscr{N}}{\tilde{C}}_{6}/({R}_{c}^{6}+{\bar{r}}^{6})$ with ${\mathscr{N}}$ the particle number, ${\tilde{C}}_{6} > 0$ the defocusing interaction strength, *R*_*c*_ the blockade radius, and $\bar{{\bf{r}}}\equiv {\bf{r}}-{\bf{r}}{\boldsymbol{^{\prime} }}$ the relative position of two particles. Let ${\rm{\Lambda }}={\mathscr{N}}{\tilde{C}}_{6}$ denote the coupling constant and when Λ is over a critical value Λ_*c*_, the 2D SF system can transition into a hexagonal SS state. Figure 1 schematically shows the ground states of the Rydberg-dressed BEC with the coupling constant below (SF state) and above (SS state) the critical value. Compared to the SF state, there are two new emerging length scales in the SS state: the lattice constant, *d*, and the radius of the SS droplets, *R*, which play important roles in QT of a SS.

The main theme of this paper is to investigate how the WT and VT behave in a SS state. Are there any new scaling laws characterizing the SS state? What roles are the new length scales playing in such a state? It is worth noting that, a self-organized structure associated with density modulation also exist in a variety of systems from nonlinear optics^42^, water waves^43^, plasma^44,45^, to planetary waves^46^. Thus current study should shed some light on the turbulence of the other systems.

As opposed to a generic quantum SS containing one or few atoms within a primitive cell, the current SS of Rydberg-dressed atomic BEC contains hundreds of atoms in a finite-size droplet within a primitive cell. Thus the system is essentially a matter-wave SS. The size of the droplets is much smaller than the lattice constant, thus the relevant wave vectors of the droplets are much larger than the inverse lattice constant. It is associated with these large wave vectors, new wave and vortex turbulence are revealed.

## Gross-Pitaevskii Equation

As mentioned earlier, Rydberg-dressed BEC is a promising system for observing the SS state^37–41^. Here, based on the Gross-Pitaevskii equation (GPE) numerical simulation, we study the quantum hydrodynamics coupled to a coherent structure in a trapped 2D Rydberg-dressed BEC.

The total energy of the Rydberg-dressed BEC can be written as the summation of kinetic, potential, and interaction energies *E*(*t*) = *E*_kin_(*t*) + *E*_pot_(*t*) + *E*_int_(*t*), where

1 $$\begin{array}{rcl}{E}_{{\rm{kin}}}(t) & = & \int \,{ {\mathcal E} }_{{\rm{kin}}}\,({\bf{r}},t)\,d{\bf{r}}=\int \,\frac{{\hslash }^{2}{|\nabla \psi ({\bf{r}},t)|}^{2}}{2m}d{\bf{r}},\\ {E}_{{\rm{pot}}}(t) & = & \int \,{V}_{{\rm{pot}}}(r){|\psi ({\bf{r}},t)|}^{2}\,d{\bf{r}},\\ {E}_{{\rm{int}}}(t) & = & \frac{1}{2}\,\int \,U(\bar{{\bf{r}}}){|\psi ({\bf{r}}^{\prime} ,t)|}^{2}{|\psi ({\bf{r}},t)|}^{2}d{\bf{r}}^{\prime} \,d{\bf{r}}.\end{array}$$

Here *V*_pot_(*r*) = *mω*^2^*r*^2^/2 is the harmonic trapping potential with *ω* the frequency and *m* the atom mass and as introduced earlier, $U(\bar{{\bf{r}}})={\mathscr{N}}{\tilde{C}}_{6}/({R}_{c}^{6}+{\bar{r}}^{6})$ is the defocusing soft-core interaction. The order parameter *ψ*, satisfying the normalization condition $\int \,{|\psi |}^{2}d{\bf{r}}=1$, is the condensate wave function. Throughout this paper, the blockade radius *R*_*c*_ and ${t}_{0}\equiv m{R}_{c}^{2}/\hslash$ are used as the units of length and time, respectively. In our simulation, a SF condensate with Thomas-Fermi (TF) radius *R*_TF_ = 6*R*_c_ was initially prepared. For the SS ground state, coupling constant is chosen to be Λ = 2400, above the critical value Λ_*c*_ = 2000. The trapping frequency is set at *ω* = 3. To generate turbulence, circulation-3 vortex and antivortex were imprinted. (Note that more initial vortex pairs have been tested and essentially lead to the same results.) Time evolution of the wave function follows the GPE: *iℏ*∂*ψ*/∂*t* = *δE*/δψ^*^. To numerically integrate GPE, we use the method of lines with spatial discretization by highly accurate Fourier pseudospectral method and the time integration is done by adaptive Runge-Kutta method of orders 4 and 5 (RK45). As resolving the trap potential can reduce the available numerical power, our simulations are performed on a square grid of 512^2^ points in a 30^2^ domain.

About the error of simulating GPE, it can be discussed by spatial and time errors of our numerical method. For spatial accuracy, we have employed Fourier-pseudospectral method, which has the property of exponential convergence compared with traditional algebraic-order-convergence methods like finite difference or finite element. High-order pseudospectral methods generally provide excellent spatial accuracy with economically practicable resolutions. For time integration, we used RK45, a time-step-adjustable Runge-Kutta method to meet the specified error tolerance, which automatically satisfies the stability criterion as well. Under these treatments and some benchmark validation, we are assured that the wave function has not been contaminated by numerical noise (round-off error) even after long-time computation.

We shall focus on the turbulence shown in the kinetic energy spectrum, *E*_kin_(*t*). As to extract the results for both WT and VT, one can separate *E*_kin_(*t*) into two mutually orthogonal parts, ${E}_{{\rm{kin}}}(t)={E}_{{\rm{kin}}}^{c}(t)+{E}_{{\rm{kin}}}^{i}(t)$, i.e., compressible and incompressible. In this regard, it is useful to express the condensate wave function *ψ*(**r**, *t*) in terms of Madelung transformation, $\psi ({\bf{r}},t)=\sqrt{n({\bf{r}},t)}\,\exp \,[i\phi ({\bf{r}},t)]$, with *n* and *φ* the density and phase, respectively. The vector field $\sqrt{n}{\bf{u}}$ with the velocity ${\bf{u}}\equiv (\hslash /m)\nabla \phi$ can then be decomposed into irrotational and solenoidal parts, or correspondingly, compressible and incompressible parts^47–50^: $\sqrt{n}{\bf{u}}={(\sqrt{n}{\bf{u}})}^{c}+{(\sqrt{n}{\bf{u}})}^{i}$, where $\nabla \times {(\sqrt{n}{\bf{u}})}^{c}=0$ and $\nabla \cdot {(\sqrt{n}{\bf{u}})}^{i}=0$. Because ${(\sqrt{n}{\bf{u}})}^{c}$ and ${(\sqrt{n}{\bf{u}})}^{i}$ are mutually orthogonal, the decomposition of the kinetic energy density follows: ${ {\mathcal E} }_{{\rm{kin}}}={ {\mathcal E} }_{{\rm{kin}}}^{c}+{ {\mathcal E} }_{{\rm{kin}}}^{i}$, where ${ {\mathcal E} }_{{\rm{kin}}}^{c,i}=(m/2)\,{|{(\sqrt{n}{\bf{u}})}^{c,i}|}^{2}$. Physically ${ {\mathcal E} }_{{\rm{kin}}}^{c}$ and ${ {\mathcal E} }_{{\rm{kin}}}^{i}$ correspond respectively to the kinetic energy densities of the sound wave and the swirls in a superflow.

To study the scaling laws of the turbulence, it is to transform ${E}_{{\rm{kin}}}^{c,i}(t)$ to the **k** space using the sum rule ${E}_{{\rm{kin}}}^{c,i}(t)={\int }_{0}^{\infty }\,{\tilde{ {\mathcal E} }}_{1D}^{i,c}\,(k,t)\,dk$. In a 2D space, ${\tilde{ {\mathcal E} }}_{1D}^{i,c}\,(k,t)$ is defined as the angle-averaged kinetic-energy spectrum

2 $${\tilde{ {\mathcal E} }}_{1D}^{i,c}(k,t)=k\,{\int }_{0}^{2\pi }\,d{\varphi }_{k}{\tilde{ {\mathcal E} }}_{{\rm{kin}}}^{i,c}({\bf{k}},t).$$

The velocity field of the superflow may change constantly, but the energy spectrum ${\tilde{ {\mathcal E} }}_{1D}^{i,c}$ will take on a stationary form as time increases.

## Scaling Laws

**Figure 2:**
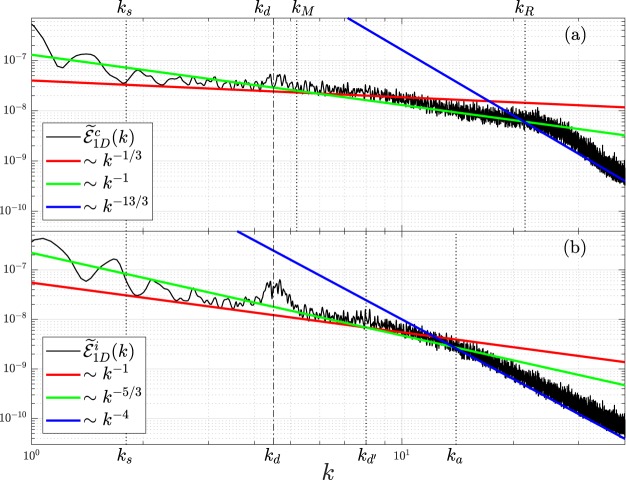
Equilibrium (**a**) compressible and (**b**) incompressible time-averaged kinetic energy spectrum obtained at time near *t* = 150. From the smallest to largest *k* scales of the system, three scaling laws are identified in both parts, with the corresponding powers noted in the legend. *k*_*s*_ and *k*_*d*_ are two common important wavevectors in both parts. *k*_*M*_ and *k*_*R*_ are two critical wavevectors in part (**a**) and *k*_*d*′_ and *k*_*a*_ are two critical wavevectors in part (**b**).

Figure 2 displays both the equilibrium compressible (top) and incompressible (bottom) parts of the time-averaged energy spectrum ${\tilde{ {\mathcal E} }}_{1D}^{i,c}(k)$ in the window of *t* = 150 ± 2.5. From the longest to shortest length scales, or the smallest to largest *k* scales, three scaling laws are identified in both parts.

### Wave turbulence

In view of Fig. 2(a) for the changes in the compressible energy, we have identified that a waveaction-cascade *k*^−1/3^ spectrum occurs at *k*_*s*_ < *k* < *k*_*M*_, which corresponds to the condensation process. An energy-cascade *k*^−1^ spectrum at *k*_*M*_ < *k* < *k*_*R*_ is also identified, which corresponds to energy transport away from the interior of the condensate through splitting into small-scale sound waves. The lower limit of the *k*^−1/3^ waveaction-cascade spectrum is *k*_*s*_ = 2*π*/*s* ≈ 1.8, where *s* ≈ 3.5 corresponds to the eventual condensate size and represents the longest length scale of the system. The upper limit of the *k*^−1/3^ scaling law is near the wavevector *k*_*M*_. As shown in Fig. 1 in the firat Brillouin zone of a 2D hexagonal lattice, ${k}_{M}=2\overline{{\rm{\Gamma }}M}=4\pi /\sqrt{3}d\approx 5.2$. The lower (upper) limit of the *k*^−1^ scaling law is near the wavevector *k*_*M*_ (*K*_*R*_), where *k*_*R*_ = 2*π*/*R* ≈ 21.5 with *R* corresponding to the radius of the SS droplet (see Fig. 1). The *k*^−1/3^ waveaction-cascade and *k*^−1^ energy-cascade are the two scaling laws commonly seen in a 2D SF BEC. Both spectra correspond to a quadratic energy dispersion *E* ~ *k*^2^ at low *k* in an *N* = 4 process^28^.

In addition to the above two scaling laws, a new *k*^−13/3^ scaling law is seen to appear uniquely for the SS state in the ultraviolet regions, starting at *k*_*R*_. By examining and comparing the compressible energy spectrum near *t* = 75 (before equilibrium) to that near *t* = 150 (after equilibrium), it is found that the ultraviolet energy spectrum increases at smaller *k*’s and decreases at larger *k*’s in the time frame. It means that the direction of energy flux in *k* space is from larger *k* to smaller *k*^28^. We thus conclude that this new −13/3 scaling corresponds to an *inverse* waveaction-cascade.

**Figure 3:**
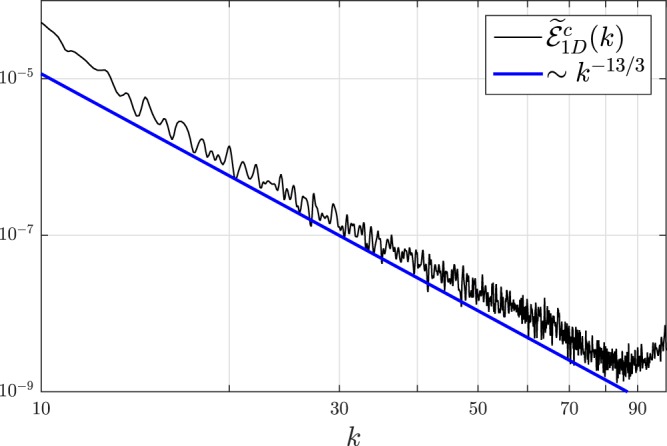
The new WT −13/3 scaling law for a 2D SS is shown to cover a large *k* range in a Rydberg-dressed BEC without the trapping potential. Same parameters are used as those in Fig. 2 except setting trapping frequency *ω* → 0. Taking into account the short-scale fluctuations near *k* = 60, the error bar on the −13/3 law is estimated to be ~0.25.

We propose that the new −13/3 scaling law is the signature in WT for a 2D SS. The lower limit of this spectrum at *k*_*R*_ is consistent with the radius of the SS droplet. The upper limit is expected to be bounded by the shortest length scale of the system, *i*.*e*., the healing length *ξ* ≈ 0.05. It will correspond to a wavevector *k*_*ξ*_ = 2*π*/*ξ* ≈ 125. In Fig. 2(a), the calculated −13/3 spectrum for a 2D trapped Rydberg-dressed BEC is only shown for wavevectors up to *k* ~ 35. Due to the effect of computing resolution, the spectrum deviates from a straight line at $k\gtrsim 50$. To verify whether this new scaling law can sustain a larger *k* range when computing resolution is increased, we perform a calculation on a similar system without the trap (by setting trapping frequency *ω* → 0). The kind of calculation enables one to simulate an infinite system by using a unit cell. In this case, the result of WT spectrum is shown in Fig. 3 for the ultraviolet regime. The *k*^−13/3^ spectrum is seen to cover almost a decade, ranging from $k\simeq 15$ to 80.

The compressible and incompressible sectors can possibly interact in the presence of the static spatial modulation. Therefore it is important to justify the new −13/3 scaling law as the value is close to the Saffman −4 law appearing in the incompressible sector [see Fig. 2(b)]. We have applied the method of least squares within the relevant scales and the line of best fits, to the results in Fig. 3, appears to have a slope −4.318. This number is consistent with the proposed −13/3. In any case, one should be more careful. The value −4.318 quoted implies the accuracy in the fourth digit and the exponent −13/3 coincides with −4.318 only up to the second digit, thus the error bar on the exponent is about 0.01 (or 2%). However, if taking into account the short-scale fluctuations happening near *k* = 60 (see Fig. 3) which covers roughly 1/4 of the order, and when comparing to best-fit line that covers about 4 orders of magnitude, the relative error of the exponent could be up to 1/16 (or 6%). Thus the error bar on the −13/3 law could be up to ~0.25. This means that the law −13/3 can well be −4.0 or −4.5 and accordingly, the appearance of the Saffman −4 law can not be excluded.

The new *k*^−13/3^ waveaction-cascade associated with the formation of SS is very distinct from the *k*^−1/3^ waveaction-cascade associated with the formation of condensate. The lower bound of the new *k*^−13/3^ scaling coincides with the radius *R* of the SS droplet, while the lower bound of the *k*^−1/3^ scaling coincides with the eventual radius *s* of the condensate.

### Vortex turbulence

**Figure 4:**
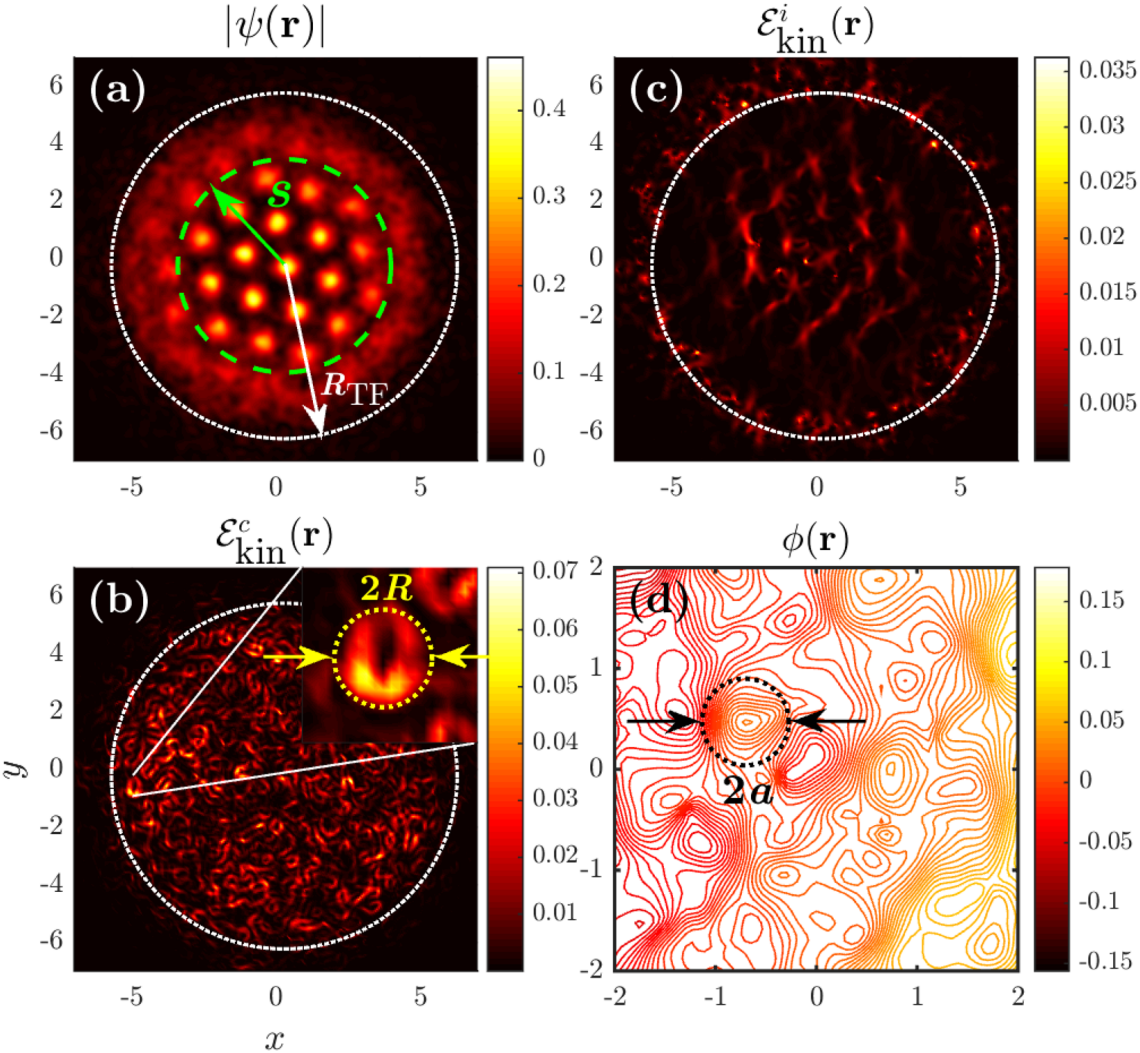
(**a**) Normalized wave function *ψ* obtained at *t* = 150. Dotted white and dashed green circles respectively indicate the initial and eventual sizes of the condensate with *R*_TF_ = 6 and *s* ≈ 3.5. (**b**) Compressible kinetic energy densities with the inset showing that the radius *R* of the SS droplet being a crucial length scale for the *k*^−13/3^ scaling law in WT. (**c**) Incompressible kinetic energy densities showing that the fluctuating vortices also from a hexagonal structure for VT. (**d**) Stream function *ϕ*(**r**) of the corresponding vector field showing that the circle of radius *a* represents the area where the fluctuating vortices mainly locate.

In the incompressible VT energy spectra displayed in Fig. 2(b), both Kolmogorov *k*^−5/3^ and Saffman *k*^−4^ scaling laws are identified. The Kolmogorov *k*^−5/3^ scaling law occurs at *k*_*d*′_ < *k* < *k*_*a*_. The lower limit *k*_*d*′_ = 2*π*/*d*′ ≈ 8 with *d*′ = *d* − 2*R* ≈ 0.8 corresponding to the gap between two adjacent SS droplets (note that when *R* → 0, *d*′ → *d*). Whereas the upper limit *k*_*a*_ = 2*π*/*a* ≈ 14 with *a* ≈ 0.45 corresponding to the radius of the maximum circle formed in the interior of the three adjacent SS droplets. The geometry gives a simple relationship between the three characteristic lengths: $d/\sqrt{3}=a+R$ or correspondingly $1/\sqrt{3}{k}_{d}=1/{k}_{a}+1/{k}_{R}$. The radius *a* also corresponds to the range over which the fluctuating vortices are mainly located [see Fig. 4(c,d)]. The lower limit of the Saffman *k*^−4^ scaling law occurs at *k*_*a*_, while the upper limit is expected to be bound by the largest *k* scale, *k*_*ξ*_, as well.

In addition to the Kolmogorov *k*^−5/3^ and Saffman *k*^−4^ scaling laws commonly seen in VT for a 2D SF BEC, we also identify a strong *k*^−1^ scaling law that covers almost a decade of the *k* range in the infrared regime. The *k*^−1^ spectrum corresponds to the motions of many independent vortices or antivortices^27,51,52^. Oscillation of the SS can constantly create vortices and antivortices in the interior, and such a fast vortex-antivortex creation and annihilation cycle results in the *k*^−1^ scaling. The lower limit of the *k*^−1^ scaling also coincides with the longest length scale *s* of the system, while the upper limit coincides with *k*_*d*′_.

Because hexagonal lattice is one of the most critical characteristics of the present system, lattice constant *d* or correspondingly wavevector *k*_*d*_ plays a crucial role in both the compressible and incompressible spectra. One sees in Fig. 2(a,b) that energy peaks rise and fall at *k* = *k*_*d*_ in both the compressible and incompressible spectra. The rise and fall of peaks are especially obvious in the incompressible one, which correspond to the creation and annihilation of vortices in the interior of the SS structure.

### Local Energy density distribution

It is also important and interesting to see how the order parameter and energy density distribute in real space. At time *t* = 150 after the equilibrium, Fig. 4(a) plots the normalized wave function (the order parameter) |Ψ(**r**)| in a square space. Both the initial and eventual sizes (*R*_TF_ = 6*R*_*c*_ and *s* ≈ 3.5*R*_*c*_) of the condensate are displayed. SS structure is seen to be robustly sustained. Figure 4(b,c) present the corresponding compressible and incompressible local energy densities, ${ {\mathcal E} }_{{\rm{kin}}}^{c}({\bf{r}})$ and ${ {\mathcal E} }_{{\rm{kin}}}^{i}({\bf{r}})$. The compressible and incompressible local energy distributions are seen to overlap strongly not only in the border area but also in the interior. The inset in Fig. 4(b) shows clearly that the radius *R* of the SS droplet plays an important role in the WT of a SS. This also explains why the lower bound of the new *k*^−13/3^ scaling law coincides with *k*_*R*_.

Because of the formation of the hexagonal SS lattice, the fluctuating vortices shown in Fig. 4(c) for VT are seen to form an analogous hexagonal structure in the interior part. That is, the density distribution of the vortex core is highly inhomogeneous. This explains why the lower bound of the Saffman *k*^−4^ scaling coincides with a short length scale *a*. To see this in more details, we plot in Fig. 4(d) the stream function *ϕ*(**r**) associated with the velocity field $\sqrt{n}{\bf{u}}$^53^. It is the solution of Poisson’s equation ${\nabla }^{2}\varphi =\hat{{\bf{z}}}\cdot \overrightarrow{{\rm{\Omega }}}$, where $\overrightarrow{{\rm{\Omega }}}=\nabla \times (\sqrt{n}{\bf{u}})$ is the 2D vorticity vector. In Fig. 4(d), the colors indicate the corresponding values of *ϕ*(**r**), and the locations and structures of eddies can be easily recognized by families of closed streamlines. From the definition of the vorticity vector $\overrightarrow{{\rm{\Omega }}}$, we note that the singular and regular parts of $\overrightarrow{{\rm{\Omega }}}$ are modulated by $\sqrt{n}$ and its gradient, respectively. As a result, the magnitude of the vorticity $\overrightarrow{{\rm{\Omega }}}$ of a vortex in high-density area is greater than that of a vortex in low-density area and, with such reasoning, we state that an eddy is energetically “larger” if it contains more circumfluent particles. Figure 4(d) clearly shows that the streams are mostly concentrated in a circle of radius *a*, which indicates that the fluctuating vortices mainly reside in this region. The figure also reveals that the streamline bundles form a (fluctuating) hexagonal structure, which is consistent with the incompressible VT energy spectra displayed in Fig. 4(c).

## On the −13/3 scaling law

It is important to investigate what causes the new WT −13/3 scaling law? As mentioned before, the −13/3 scaling law corresponds to an inverse waveaction cascade, so we proceed to search for what wave is responsible for. Let us first consider a wave that has an effective dispersion *ω*_*k*_ ~ *λk*^*α*^ for certain *k* range, where *λ* is a positive parameter and *α* is the power. The dimension of *λ* is thus [*λ*] = [*t*]^−1^[*l*]^*α*^ with *l* (*t*) denoting the length (time) scale. The energy balance equation reads as ${\partial }_{t}{\tilde{ {\mathcal E} }}_{1D}(k)+{\partial }_{k}\varepsilon =0$, where ${\tilde{ {\mathcal E} }}_{1D}$ is the local (compressible) kinetic-energy density spectrum [see Eq. (2)] and *ε* is the corresponding energy flux. Besides, ${\tilde{ {\mathcal E} }}_{1D}$ is related to the total (compressible) kinetic energy density *E* via $E=\int \,{\tilde{ {\mathcal E} }}_{1D}\,dk$. The dimensions of *E*, ${\tilde{ {\mathcal E} }}_{1D}$, and *ε* are thus [*E*] = [*l*]^5−*D*^[*t*]^−2^, $[{\tilde{ {\mathcal E} }}_{1D}]={[l]}^{6-D}{[t]}^{-2}$, and [*ε*] = [*l*]^5−*D*^[*t*]^−3^, respectively with *D* the dimension of the system in real space^28^. In an *N*–wave process, one also has the rate relation, $\varepsilon \sim \dot{E}\sim {E}^{N-1}$.

In addition to the conserved energy, for an even number *N*–wave process (as for the GPE simulation, *N* = 4), the total waveaction or “particle number”, $\int \,{\tilde{ {\mathcal E} }}_{1D}/{\omega }_{k}\,dk\equiv \int \,{n}_{k}\,dk$, is also conserved for the *N*/2 → *N*/2 processes. Thus one can also have the following waveaction balance equation, ∂_*t*_*n*_*k*_ + ∂_*k*_*ζ* = 0, where *ζ* = *ε*/*ω*_*k*_ is the waveaction flux. The dimensions of *n*_*k*_ and *ζ* are [*n*_*k*_] = [*l*]^6−*D*^[*t*]^−1^ and [*ζ*] = [*l*]^5−*D*^[*t*]^−2^, respectively. Analogous to that for *ε*, one also has a rate relation for *ζ*, *ζ* ~ *E*^*N*−1^. With the above dimension analyses, one can come up the following scaling law for the energy spectrum associated with the conservation of waveactions: ${\tilde{ {\mathcal E} }}_{1D}\sim {\lambda }^{x}{\zeta }^{1/(N-1)}{k}^{y}$. Explicitly^28^

3 $$x=2-\frac{2}{N-1}\,{\rm{and}}\,y=D-6+\frac{5-D+2\alpha (N-2)}{N-1}.$$

Substitution of *N* = 4 into the first equation of (3) gives *x* = 4/3. More interestingly, substitution of *D* = 2, *N* = 4, and the identified power *y* = −13/3 into the second equation of (3) gives *α* = −1. This indicates that the wave which causes the new WT −13/3 scaling law has an effective dispersion with a minus-one power at the relevant scales:

4 $${\omega }_{k}\sim \lambda {k}^{-1}.$$

**Figure 5:**
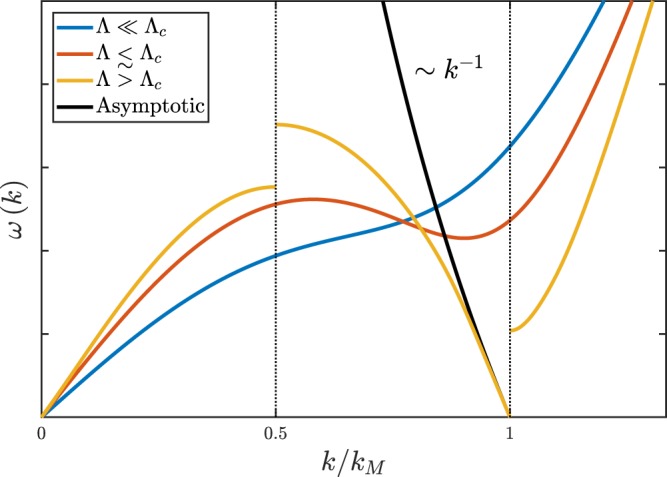
Blue and red curves plot the elementary excitation spectra *ω*(*k*) for the SF state at ${\rm{\Lambda }}\ll {{\rm{\Lambda }}}_{c}$ and ${\rm{\Lambda }}\lesssim {{\rm{\Lambda }}}_{c}$ respectively, obtained from (5). Orange curve plots the longitudinal elementary excitation spectrum *ω*(*k*) along Γ-*M* direction (see Fig. 1) for the hexagonal SS state at Λ > Λ_*c*_, obtained by solving the BdG equation. Black line displays the *k*^−1^ asymptotic behaviors of the orange curve at $k\to {k}_{M}^{-}$.

It is of great interest if one can solve the entire wave dispersion for the current Rydberg-dressed BEC system and confirm that *ω*_*k*_ ~ *λk*^−1^ for *k* ~ *k*_*R*_. This is not feasible, however. We instead seek for ideas from the elementary excitation to which analytical results are available for the system^32^. In the absence of the trapping potential, the *stable* elementary excitation of the system in the SF state (when coupling constant Λ < Λ_*c*_) behaves as

5 $$\hslash \omega (k)=\sqrt{\frac{{\hslash }^{2}{k}^{2}}{2m}\,[\frac{{\hslash }^{2}{k}^{2}}{2m}+2{n}_{0}\tilde{U}(k)]},$$

where *n*_0_ is the uniform density and $\tilde{U}(k)=\tilde{U}(|{\bf{k}}|)$ is the Fourier transformation of the isotropic soft-core interaction function *U*(**r**) introduced earlier. Since $\tilde{U}(k)$ is negative in certain *k* intervals, dispersion (5) could become *unstable* when Λ > Λ_*c*_. It means that above the critical point, an instability, named “roton instability”, occurs and the SF state will transition into a periodic SS state. In Fig. 5, based on Eq. (5) the blue and red curves plot the elementary excitation spectra *ω*(*k*) for the stable SF state at ${\rm{\Lambda }}\ll {{\rm{\Lambda }}}_{c}$ and ${\rm{\Lambda }}\lesssim {{\rm{\Lambda }}}_{c}$, respectively. Whereas orange curve plots the longitudinal elementary excitation spectrum *ω*(*k*) for the periodic hexagonal SS state at Λ > Λ_*c*_ which is obtained by solving the Bogoliubov-de Gennes (BdG) equation. Of most interest, a low-energy gapless mode is seen to appear at $k\to {k}_{M}^{-}$ for the SS state, where *k*_*M*_ is defined in Fig. 1. As displayed by the black line in Fig. 5, the asymptotic behavior of the orange curve at $k\to {k}_{M}^{-}$ does exhibit a minus-one power dispersion, *ω*(*k*) ~ *k*^−1^.

The low-energy gapless *k*^−1^ elementary excitation does not have a direct relation to the new −13/3 WT scaling law though. One reason is that the low-energy gapless *k*^−1^ elementary excitation occurs at *k*_*M*_, while the *k*^−1^ wave dispersion that causes the new −13/3 WT scaling law occurs at *k*_*R*_. Secondly, the wave that causes the new −13/3 WT scaling law should have relatively high energy, while the *k*^−1^ elementary excitation is the low-energy one. Nevertheless, it still shed light on why in the SS state, the wave dispersion behaves ~*k*^−1^ in the ultraviolet regions. When Λ > Λ_*c*_ in the SS state, negative part of $\tilde{U}(k)$ can result in a significant reduction in the energy spectrum. The free-particle wave dispersion *k*^2^ for the kinetic energy can be significantly reduced and consequently a down-turn *ω*(*k*) can occur. A down-turn dispersion implies a negative power. As for why the new −13/3 WT scaling law coincides with *k*_*R*_, it can be understood in this way. When SS forms, the condensed atoms pile up in a lattice of droplets. Thus the ultraviolet waves will conform to the droplets with an onset wavevector *k*_*R*_.

## Conclusion

In summary, quantum turbulence including both wave and vortex dynamics is investigated in a 2D trapped Rydberg-dressed Supersolid (SS). The SS state of the Rydberg-dressed BEC occurs when the coupling constant is over a critical point. Of most interest, we discover a new −13/3 scaling law in the wave turbulence, which is the signature for the 2D SS state. This −13/3 scaling implies that the wave has an effective *k*^−1^ dispersion at the wavevector consistent with the radius of the SS droplets. As self-organized structure associated with density modulation can exist in a variety of systems from nonlinear optics^42^, water waves^43^, plasma^44,45^, to planetary waves^46^, our study should shed some light on the wave turbulence of other systems.
